# Health professional perceptions of a pharmacist-led interprofessional chronic pain clinic

**DOI:** 10.1177/17151635231213326

**Published:** 2023-11-28

**Authors:** Derek Jorgenson, Katelyn Halpape, Michael Siton

**Affiliations:** College of Pharmacy and Nutrition, University of Saskatchewan. Saskatoon, Saskatchewan

## Background

Managing patients with chronic non-cancer pain (CNCP) poses challenges for health professionals. Each patient’s experience with CNCP is unique and this subjectivity makes it difficult to objectively assess and monitor the condition.^1^ CNCP can have multiple underlying causes and diagnosing the etiology can be difficult, relying heavily on patient-reported symptoms, a detailed evaluation of the pain history and a clinical assessment.^1^ Unlike many other medical conditions, CNCP often lacks specific diagnostic tests to confirm the cause, or even the existence of the pain, further complicating management for health professionals. Not only does CNCP have a significant impact on an individual’s physical health and function, but it can also impact emotional, mental and spiritual health and well-being.^2^ Thus, optimal CNCP management requires holistic care.^1-3^

Goals of therapy for people with CNCP vary significantly and it can be time-consuming for health professionals to develop goals alongside patients.^4,5^ This is a challenge for health providers with large caseloads and limited patient contact time. Finding the safest and most effective treatment options also often requires a laborious trial-and-error process.^6^ The opioid crisis and the recent awareness that opioids often cause more harm than benefit in CNCP treatment has created a shift in practice guidelines, where opioids are no longer at the forefront of treatment.^3,7,8^ CNCP is also frequently complicated by comorbid diagnoses of mental health conditions, such as depression, anxiety and personality disorders, which often require referrals to specialists and complicates pharmacotherapy, especially optimization of opioid pharmacotherapy.^2,9^

As a result of these challenges, CNCP management requires significant time and resources, making it difficult, if not impossible, for any health professional to manage independently.^1^ Referring individuals living with CNCP to interprofessional chronic pain clinics is the obvious solution to this dilemma and is the route recommended in guidelines.^3^ Unfortunately, in Canada, there is a shortage of these comprehensive interprofessional chronic pain clinics, especially in rural regions.^1^

The University of Saskatchewan Chronic Pain Clinic (UCPC) opened in March 2020 to improve access to interprofessional CNCP care by utilizing a unique pharmacist-led, interprofessional team approach.^10^ In addition to treating individual patients with CNCP, the UCPC aims to increase the capacity of primary care practitioners to manage CNCP by improving their knowledge and understanding of CNCP management. The team includes 2 full-time equivalent (FTE) of pharmacists, 2 FTE of social workers, 2 FTE physical therapists and 0.4 FTE of physicians. To access UCPC services, patients can be referred by any health professional or can self-refer, however, they are required to have a prescribing physician or nurse practitioner (NP) who is willing to collaborate with the UCPC for medication changes, as the UCPC does not prescribe.^11^ The UCPC pharmacist acts as a patient navigator and takes responsibility for communicating with the patient’s physician or NP, including providing support for implementing changes to pain medications. Most patients have multiple individual and/or group appointments (virtually or in-person) with the UCPC pharmacist, social worker and physical therapist. The UCPC physician rarely interacts directly with patients but is a clinical resource to the UCPC team to discuss challenging cases (by videoconference). Additionally, the UCPC offers a referring health provider mentorship program. After referring a patient, health providers are offered one-on-one discussion(s) with the UCPC pharmacist and physician, who provide patient-specific mentorship and education on CNCP management and/or opioid prescribing. The UCPC also delivers group education classes on various topics for patients and caregivers. The UCPC attempts to discharge patients back into the care of their physician or NP within 6 months.

The UCPC team determined that it was important to elicit feedback from referring health care professionals to evaluate the program. The purpose of this study was to describe the experiences of the health professionals who collaborated with the UCPC in the shared management of patients with CNCP.

## Methods

This study used a paper-based postal survey with the primary outcome of identifying aspects of the UCPC that collaborating health professionals found valuable, along with suggestions for improvement. Any health professional that referred a patient who attended at least one appointment at the UCPC between January and December 2021 was included in the study. For patients who self-referred to the UCPC, the primary prescriber listed on the referral form was included in the study. The questionnaire that was mailed to participants included 8 questions collecting demographic information, 10 Likert scale questions collecting information about the health professional’s perceptions and experiences with the UCPC and 2 open-ended questions that elicited free-text responses regarding what respondents liked best about the program and what could be improved. The questionnaire was developed based on previously published studies and utilizing the expertise of the UCPC external advisory committee.^12,13^ The questionnaire was pre-tested on 5 family physicians, 1 physical therapist and 2 pharmacists who did not meet the inclusion criteria of the study. The questionnaire took less than 5 minutes to complete in the pre-test.

Questionnaires were mailed to potential participants approximately 3 months after their patient attended an initial appointment at the UCPC, when it was expected that the health professional and their patient experienced the program’s services. Surveys included self-addressed stamped envelopes to return completed questionaries. Health professionals who returned a questionnaire were entered in a draw for a $100 gift card as an incentive to participate. Quantitative data (i.e., answers to the Likert scale questions) were entered into a Microsoft Excel spreadsheet and analyzed using descriptive statistics. Qualitative data from the open-ended questions were analyzed using thematic analysis by the Canadian Hub for Applied and Social Research at the University of Saskatchewan, which has extensive experience with this methodology. The protocol was approved by the University of Saskatchewan Research Ethics Board.

## Results

A total of 31.6% (n = 31/98) of eligible participants responded to the survey. The participants included physicians (77.4%, n = 24/31), nurse practitioners (12.9%, n = 4/31), physical therapists (6.5%, n = 2/31) and one pharmacist. Almost 60% of respondents (n = 18/31) were 10 years or less into their practice, with 35.5% (n = 11/31) being more experienced (i.e., ≥16 years in practice). Approximately half of respondents (n = 16/31) practiced in areas with a population over 100,000 people, while just over one-third (n = 11/31) were working in rural areas with a population under 10,000. Most respondents referred a patient to the UCPC, with only 4 physicians (12.9%) reporting that their patient self-referred to the program.

**Figure 1:**
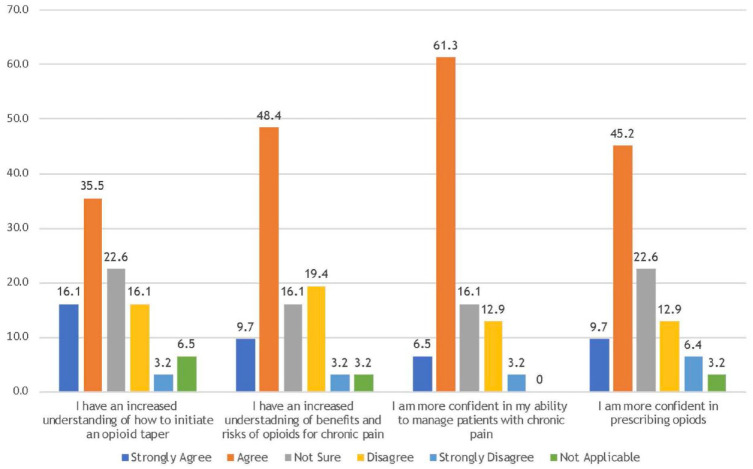
Impact on health provider confidence and understanding of chronic pain care, % (n = 31)

Respondents were asked about the extent to which they believed that their interactions with the UCPC, through their patient’s referral, improved their confidence and understanding of various aspects of CNCP management (Figure 1). Just over half (54.9%, n = 17/31) of respondents strongly agreed or agreed that since interacting with the UCPC, they are more confident in prescribing opioids. Almost 70% (n = 21/31) strongly agreed or agreed that they are more confident in their ability to manage patients with chronic pain and over half (51.6%, n = 16/31) strongly agreed or agreed that they had an increased understanding of how to initiate an opioid taper. Finally, 58.1% (n = 18/31) strongly agreed or agreed that they had an increased understanding of the benefits and risks of using opioids to treat CNCP (Figure 1).

**Figure 2:**
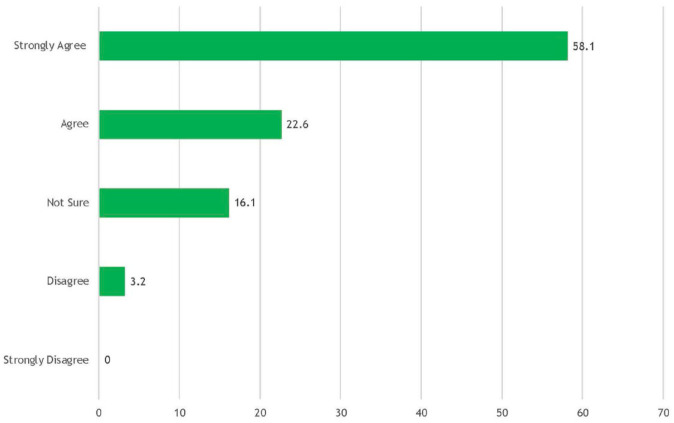
The USask chronic pain clinic helped me with the management of my patient % (n = 31)

When asked about their satisfaction and overall experiences with the clinic, 90.3% (n = 28/31) strongly agreed or agreed that the UCPC was a useful resource to their practice and 93.5% (n = 29/31) strongly agreed or agreed that the recommendations and consultation letters from the UCPC team were helpful. When asked about the impact of the clinic on their patients, 80.7% (n = 25/31) strongly agreed or agreed that the UCPC helped them with the management of their patients (Figure 2). Finally, 90.3% (n = 28/31) of respondents reported that they would recommend the UCPC to colleagues.

The open-ended questions elicited many comments regarding what the health professionals liked about the clinic. Overall, the clinic was praised for its communication between the UCPC team and patients, but also between the UCPC team and the respondents. Communication was commonly described as timely and efficient. Many respondents also stated that the program offers good patient care, valuable self-management education and quality recommendations regarding managing pain medications. There were also several comments stating that it was beneficial for the patient to hear consistent messages from the respondents and the UCPC team regarding the management of their pain.

Comments related to what could be improved included better advertising the service so that more health providers were aware of how to refer a patient. Several physician respondents also requested that the UCPC not require any test results (e.g., urine drug screen) as part of the referral form and some suggested that it would be helpful to have the UCPC physician prescribe when a medication change was recommended by the team.

## Discussion

Previous research has consistently described the benefits of interprofessional chronic pain clinics, including the model of care used at the UCPC.^11,14,15^ This health professional experience survey adds valuable data to the existing literature. One of the goals of the model of care used at the UCPC is to increase the capacity of the existing primary care system to better manage CNCP, aiming to reducing reliance on specialty services such as interprofessional chronic pain clinics. The results of this study suggest that the UCPC was at least partially successful in achieving this aim. The majority of respondents agreed that their interactions with the UCPC improved various aspects of their CNCP care, such as prescribing opioids, managing chronic pain, opioid tapering and understanding the risks and benefits of prescribing opioids (Figure 1). This is a key result because it is unsustainable for a health system to constantly add new specialty services to fill gaps in care and there is value in embracing innovations in health service delivery that also increase the capacity of the existing primary care system. The results of this study do not explain what aspects of the UCPC model of care contributed most to this finding. However, the health provider mentorship program described in the methods section was created and implemented by the UCPC team intentionally for this purpose.

The primary aim of the UCPC is to provide high-quality CNCP care by assisting patients achieve their specific goals of therapy, using a combination of mind, movement and medication-based therapies. The findings of this study provide some preliminary evidence that this aim is being achieved. When asked about the impact of the clinic, most respondents agreed that the UCPC helped them with the management of their patients (Figure 2). In addition, the free text responses focused heavily on the strong and consistent communication that exists between the UCPC, the patient and the primary care providers. These findings do not provide evidence of direct impact on patient outcomes, but they do offer a signal that primary care providers were better equipped to provide care to these patients as a result of the UCPC.

The results of this study also describe a cohort of referring health providers who were very satisfied with the service. Possibly the best evidence of this conclusion is the finding that over 90% of respondents stated that they would recommend the UCPC to colleagues. This is an important finding because the model of care used at the UCPC is quite different compared to typical physician-led chronic pain clinics. If this pharmacist-led interprofessional model was not acceptable to other health providers, who must refer patients and collaborate with the UCPC team for care plan implementation, the service would not be sustainable.

The results of this study are consistent with previously published research on the UCPC model of care, which strengthens the trustworthiness of the findings.^11^ However, there are limitations to the data that affect the external validity and generalizability of the results. The questionnaire was completed by a relatively small number of health professionals and the response rate was below 40%, which is not optimal, but is also typical of health professional surveys. There is also a risk of several forms of bias inherent to survey research. This includes non-response bias, in that the views of those who did not respond are likely different than those who responded. The questionnaire wording may also have led to acquiescence bias for some items because respondents may have a tendency to agree with the questions that are not worded neutrally. There is also a risk of social desirability bias, where the respondents may believe that it would be more favourable if they answered positively about items that might question their skills or competence (e.g., understanding of opioid management). Additionally, the majority of respondents were physicians or NPs, thus, the generalizability of the results to other health professionals is limited. The study was also completed in a single clinical site, suggesting that the results may not translate to other regions of the world and future studies are necessary to confirm the findings.

## Conclusion

This health professional survey found that the pharmacist-led interprofessional model of CNCP care used by the UCPC resulted in improvements in health provider’s confidence in delivering CNCP care, while providing a specialty service that was highly appreciated by the health providers. This data suggests that this unique team-based model for CNCP management is a viable alternative to existing physician-led models. Future research should aim to confirm these findings, preferably using a randomized, controlled trial that includes patient outcome data. ■
